# Ascertainment of Community Exposure Sites to Ross River Virus During the 2020 Outbreak in Brisbane, Australia

**DOI:** 10.1093/infdis/jiae578

**Published:** 2024-11-26

**Authors:** Tatiana Proboste, Damber Bista, Nicholas J Clark, Sahil Arora, Gregor Devine, Jonathan M Darbro, Deena S Malloy, Daniel Francis, Ricardo J Soares Magalhães

**Affiliations:** Queensland Alliance for One Health Sciences, School of Veterinary Science, University of Queensland, Gatton, Queensland, Australia; Institute for Life Sciences and the Environment, University of Southern Queensland, Toowoomba, Queensland, Australia; Queensland Alliance for One Health Sciences, School of Veterinary Science, University of Queensland, Gatton, Queensland, Australia; Queensland Alliance for One Health Sciences, School of Veterinary Science, University of Queensland, Gatton, Queensland, Australia; Mosquito Control Laboratory, QIMR Berghofer Medical Research Institute, Brisbane, Queensland, Australia; Metro North Public Health Unit, Brisbane, Queensland, Australia; Metro North Public Health Unit, Brisbane, Queensland, Australia; Metro North Public Health Unit, Brisbane, Queensland, Australia; Queensland Alliance for One Health Sciences, School of Veterinary Science, University of Queensland, Gatton, Queensland, Australia; Children's Health Research Centre, Children’s Health and Environment Program, The University of Queensland, South Brisbane, Queensland, Australia

**Keywords:** Ross River virus, spatiotemporal analysis, vector-borne disease, network analysis, cluster analysis

## Abstract

This study investigated potential Ross River virus (RRV) exposure sites in Greater Brisbane during the Queensland coronavirus disease 2019 lockdown (January–July 2020). Using RRV notifications, cluster identification techniques, and mobile phone data for movement network analysis, the study examined 993 RRV cases and 9 million movement trajectories from residential RRV cluster areas (hot spots). The findings revealed that population movement was a key risk factor to RRV incidence within hot spots, whereby highly interconnected areas had more RRV cases during lockdown. While environmental conditions within RRV hot spots were less significant compared with their connectivity, areas with higher vegetation density had fewer RRV cases. The study also noted that individuals from RRV hot spots spent less time in green areas before lockdown than during and after lockdown. The results suggest that population movement significantly influenced the 2020 RRV outbreak. These insights can help adapt current vector control and surveillance protocols to target areas identified in this study.

## Teaser

During the coronavirus disease 2019 lockdown, highly connected areas and population movement were risk factors for Ross River virus spread in Brisbane, Australia. Usually, areas with lush vegetation had higher RRV incidence, but during the lockdown we found that this link saturates with very high vegetation cover leadin to less cases, highlighting the importance of connectivity and environmental factors in disease transmission.

Ross River virus (RRV) infection is the most incident arboviral disease in Australia [1], with approximately 5000 cases reported annually [2]. The disease has an incubation period that varies from 7 to 9 days [3]. Even though most cases are asymptomatic, infection can result in prolonged and debilitating disease that includes headaches, lethargy, rash, fever, and muscle pain [4, 5], with no vaccination or specific treatment available [6]. Despite the significant human health burden, the transmission cycle of RRV and associated ecological risk factors are not fully understood.

The relationship between vectors and RRV hosts is complex and changes across landscapes [7]. Exposure and/or infection to RRV has been detected in >40 species of mosquitoes [1] and a range of multiple vertebrate hosts, including horses, macropods, opossums, and humans [8, 9]. The abundance of RRV-infected mosquitoes has been shown to be linked to weather variables, such as rainfall, humidity, and temperature [10, 11]. In Australia, the state of Queensland has favorable weather conditions for mosquito breeding and thus disease transmission [12]. Moreover, health education of the at-risk population may also influence disease transmission [2], particularly people's vector protection behaviors that might reduce exposure to infected mosquitos in high-risk areas. Human behavior is key in the dynamics of vector-borne disease spread [13].

During the 2020 coronavirus disease 2019 (COVID-19) lockdown in Australia, one of the largest RRV outbreaks occurred, with >1100 cases. The COVID-19 response in Brisbane, initiated in March 2020, included public health orders on restriction on movement, gatherings, and business activities, which were implemented throughout Queensland, followed by closure of all nonessential businesses from April 2020. These public health orders were then lifted on 2 May 2020 [14, 15]. The restriction of population movement provides a unique opportunity to study disease exposure in contexts where the timing and location of exposure are difficult to ascertain. For example, a significant proportion of the RRV cases reported in April 2020 may have been involved exposure to RRV-infected mosquitoes at the start of the lockdown, near residences or within lockdown areas. If RRV's main host is nonurban wildlife, we would have expected fewer city cases or more periurban clusters due to movement restrictions.

The current research aimed to investigate risk factors for RRV exposure during the COVID-19 lockdown in Brisbane, Australia, contributing to a broader understanding of vector-borne disease and human behavior. By identifying potential locations visited by populations within RRV hot spots and quantifying the characteristics of such locations, this study provides insights extending beyond RRV and Brisbane. To achieve the study aim, we carried out our investigations with 3 objectives. First, we quantified the relationship between the weekly incidence of RRV notifications and temporally variant factors (rain, temperature, and time spent outdoors). Second, we investigated the association between the incidence of RRV notifications and environmental variables and population movement of known RRV geospatial clusters. Finally, we identified the locations frequented by the population from RRV clusters.

## METHODS

This research uses an ecological spatiotemporal time-series study design to identify putative exposure sites for RRV infections across councils of the Metro North and Metro South Public Health Unit during the COVID-19 social distancing period of January to July 2020 (details in the Supplementary materials).

### RRV Data Source

We obtained and analyzed the 993 georeferenced RRV notification data from the Notifiable Conditions System (NOCS), reported during the period from 1 January to 31 July 2020; records were approved for release by NOCS on 20 July 2021.

**Figure 1.**
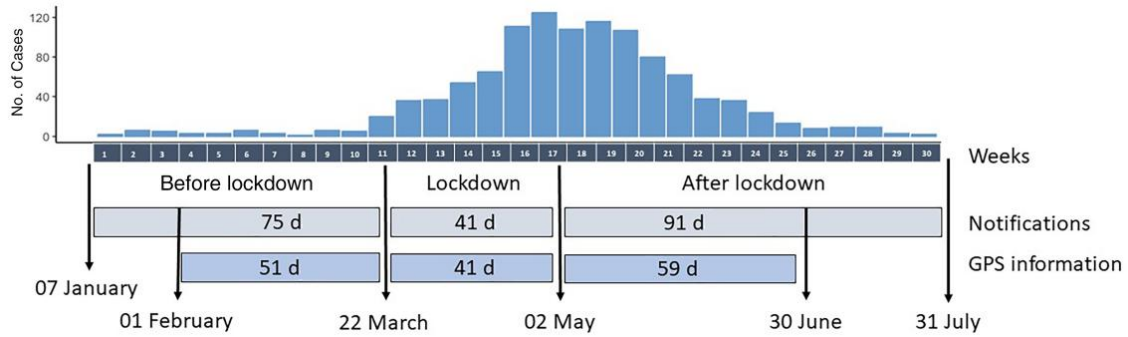
Diagram of the study period with dates of the prelockdown, lockdown, and postlockdown periods. Abbreviation: GPS, global positioning system.

The variables included in the query included notification date, onset date, age, sex, and address (details in the Supplementary materials). Population data at the mesh-block level (mesh blocks are the smallest geographic unit in Australia) was obtained from census data from the Australian Bureau of Statistics for 2016. The data were divided into 3 periods: before lockdown (7 January to 22 March 2020, when RRV cases started but no movement restrictions were in place), lockdown (23 March to 2 May 2020, when movement restrictions were in place), and after lockdown (3 May to 31 July 2020, after movement restrictions were lifted), as the behavior of the community is expected to change due to the movement restriction across the 3 times period (Figure 1).

### Environmental Data

We obtained environmental data affecting mosquito density from the Queensland government Long Paddock. This included rainfall per week (in millimeters) and average maximum temperature (in degrees Celsius) per week. We also included distance to water bodies, distance to green areas, and the foliage projective cover, which represents the percentage of vegetation cover (see the Supplementary materials).

### Population Movement Data and Data Processing Steps

We retrieved third-party deidentified mobile phone data from the Output AI industry-partner company, which contained the longitude and latitude of each signal. We used this information to quantify changes in community mobility in response to COVID-19 social distancing restrictions We estimated people's location by using mobile phone global positioning system (GPS) records (see the Supplementary materials).

To determine the movement of people living in the clusters, we created a network model of community mobility. We investigated the movement patterns of people inhabiting the cluster area. We used the assumed household as the starting point, and only the people that were identified to have the highest number of points within RRV clusters were included in this analysis. All analyses were conducted at the mesh-block level.

We limited trajectory analyses to tracks <1000 m/h, to exclude car travel, focusing on higher-risk activities like walking or spending time outdoors, where exposure to mosquito bites is more likely. We analyzed the trajectory for each period using the adehabitat package [17] and estimated the mean distance travelled per day and the total time (minutes) spent in each green area and divided by the number of people who visited the area. In addition, we divided the day into 4 groups: morning (6–10 Am), day (10 Am to 4 Pm), evening (4–7 Pm), and night (7 Pm to 6 Am). Then, using the adehabitat package, we estimated the spatial kernel density across the diel cycle.

### Spatiotemporal RRV Cluster Analyses

To analyze the clustering patterns of RRV notifications we used the Moran *I* statistic to assess spatial clustering and the local Moran *I* statistic as an indicator of spatial association of the RRV clusters in the study area. We used 999 permutations to estimate pseudo-*P* values and *z* values using GeoDa [18]. A *z* score is generated by the local Moran *I* statistic to determine the significance level of clusters. Surroundings with spatial clusters will be indicated by a high positive *z* score, and the presence of spatial outliers will be represented by a low negative *z* score. The geographic unit of analysis was the geographic center of the mesh block. We defined a community cluster as ≥2 cases associated within a 5-km radius and a time aggregation of a month (1 March to 31 May 2020).

### Association Between RRV Notification Incidence and Temporal Variant

We calculated cross-correlation between weekly RRV notifications and environmental covariates rainfall per week (in millimeters) and average maximum temperature (in degrees Celsius) per week using the *stats* package in R software (version 4) [19].

### Association Between RRV Notification Incidence, Environmental Data, and Population Movement Network Measures

We explored the association between the number of RRV notifications, 3 environmental variables (green areas, distance to water bodies, and vegetation density), and network measures (centrality, betweenness and degree) using a generalized additive model with Poisson family and log link function (see the Supplementary materials). We created buffers around each cluster based on the average distance people traveled in different periods. This was done to include relevant information in our models, as clusters were dispersed and changed location over time. The buffer distances were 910, 740, and 880 m for the periods before, during, and after the lockdown respectively, reflecting the average daily distances covered.

The generalized additive model was built using the number of RRV cases in each mesh block in each period as our outcome variable. We included several time-varying penalized smooth functions of environmental and demographic covariates to ask how their associations with the log number of RRV cases varied across the 3 time periods (see the Supplementary materials).

### Relation Between Population Movement Network Measures and Landscape

We calculated the average time spent in green areas by people per week during each period using the outputs from the trajectory analysis. We calculated cross-correlation between weekly RRV notifications and average weekly distance (in kilometers), and the average time spent walking outdoors per week.

### Ethical Approval

This project (ID 71859 LNR/2021/QRBW/71859) received ethical approval from Royal Brisbane and Women’s Hospital (HREC 71859) on 15 March 2021, research ethics ratification from The University of Queensland (2021/HE000937) on 21 April 2021, and Public Health Act authorization to request health information held (PHA 71859).

## RESULTS

### Seasonal and Demographic Trends in RRV Cases

We found that the peak in the incidence of RRV notification was in April and May for the study area, and the age distribution suggested higher exposure or susceptibility among certain age groups as cases follow a normal distribution (Supplementary Figure 1).

### Impact of Lockdown on Population Movement and Exposure Risk

**Figure 2.**
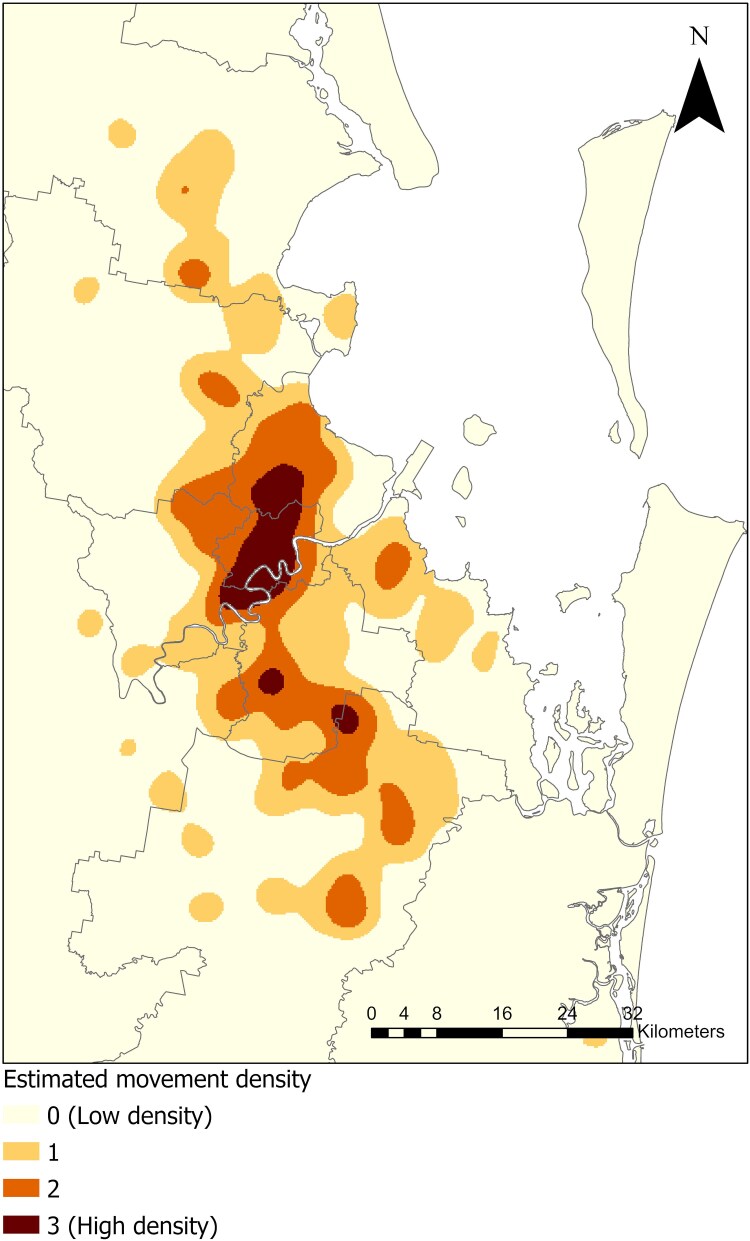
Kernel density of people's movement.

During lockdown, people traveled shorter distance, with a mean (SD) of 0.74 (0.74) km, based on 1052 unique devices. The mean (SD) distance travelled by people during the prelockdown period was 0.91 (0.8) km, comprising records from 736 unique devices; in the postlockdown period, we estimated a mean (SD) distance of 0.88 (0.7) km, based on 1062 unique devices. We also mapped the movement density for the whole study period (Figure 2) and based on the time of the day to see differences between times, as mosquito bites are more likely to occur during the morning and the evenings for most mosquito species (Supplementary Figure 2).

### Identifying High-Risk Areas for Targeted Interventions

**Figure 3.**
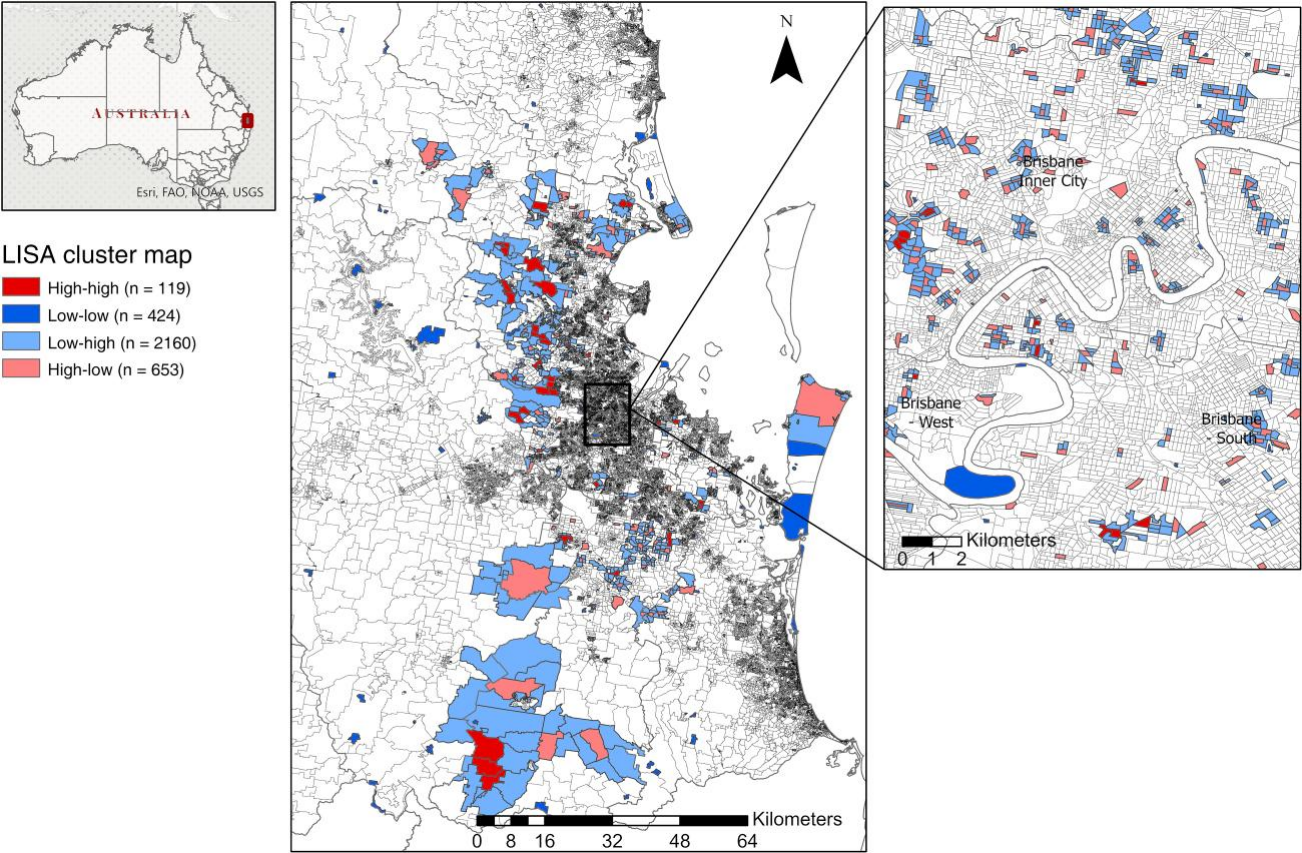
Local Indicator of Spatial Association (LISA) cluster map for the accumulative Ross River virus reports across councils of the Metro North and Metro South public health units between 1 January and 31 July 2020.

Spatial analysis identified 119 RRV clusters, or hot spots, for the whole study period, which represent the areas with high incidence of cases that are close to other areas with high incidence of RRV notifications (high-high), 424 mesh blocks that were identified as low-low or “cold spots.” We identified 2160 mesh blocks classified as low-high and 653 classified as high-low (Figure 3). The overall Moran *I* statistic (from January to July) was .005, the estimated pseudo-*P* value was .025, and the *z* value was 1.8671.

### Environmental and Network Influence on RRV Transmission

**Figure 4.**
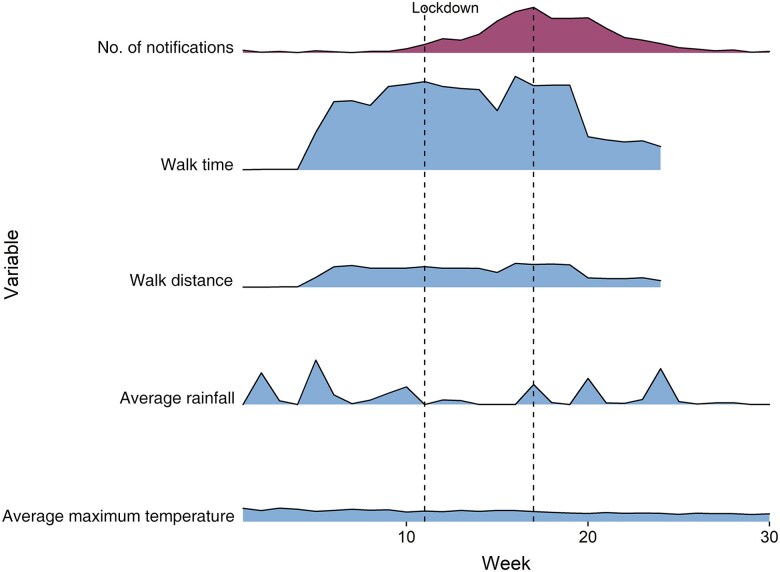
Distribution of number of Ross River virus notifications per week, average walk time, average walk distance per week, average rainfall per week, and average maximum temperature.

While in our cross-correlation analysis (Supplementary Figure 4), we can see a 5-week lag between weekly rainfall and the increase in RRV notifications, this was not statistically significant, nor was the association between average maximum temperature per week and RRV cases (Figure 4).

**Figure 5.**
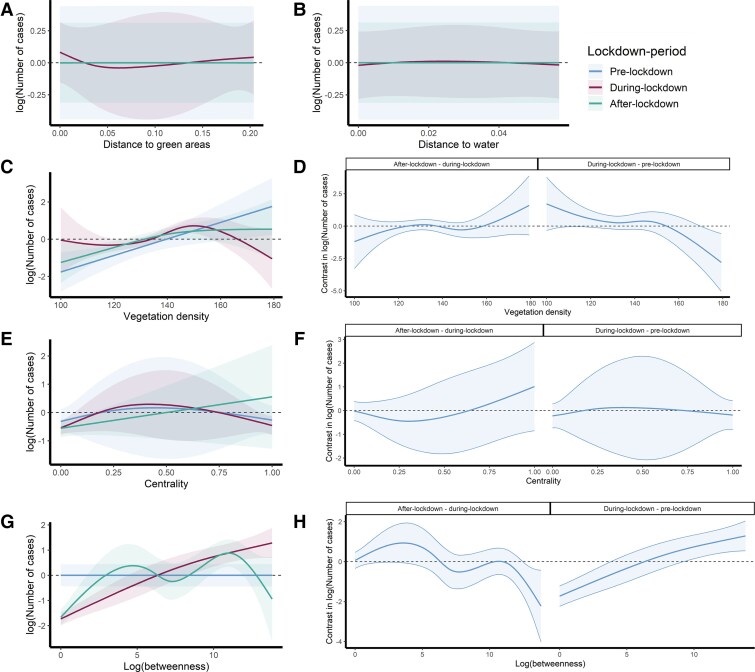
*A–C, E, G,* Plots of conditional smooth effects of the log number of Ross River virus cases predicted from a generalized additive model with the following responding variables: distance to green areas (*A*), distance to water (*B*), vegetation density (*C*), centrality (*E*), and betweenness (*G*). *D, F, H,* Comparison of smooth effects showing changes in expected functional relationships over time for vegetation density (*D*), centrality (*F*), and betweenness (*H*). All other predictors were held at 0 when calculating conditional effects of focal predictors.

Our generalized additive model found support for time-varying nonlinear effects of environmental and network predictors, with particularly important effects of vegetation density and betweenness (Figure 5). Vegetation density (Figure 5*C*) was estimated to have a strong positive linear effect before lockdown, but this effect was dampened toward a weaker nonlinear effect during lockdown and postlockdown periods. For betweenness, the model estimated a flat, unimportant function during the prelockdown period (Figure 5*G*). But this effect changed substantially during the lockdown period, when betweenness was estimated to be highly important predictor with an almost linear positive effect. The effect was then dampened back toward a weaker nonlinear function after lockdown. All other effects were found to be unimportant, with the model regularizing them to flat functions (Figure 5). For information about the spatial correlation across time for the individual's smooth effect, please refer to the Supplementary materials (Supplementary Figures 5 and 6).

**Figure 6.**
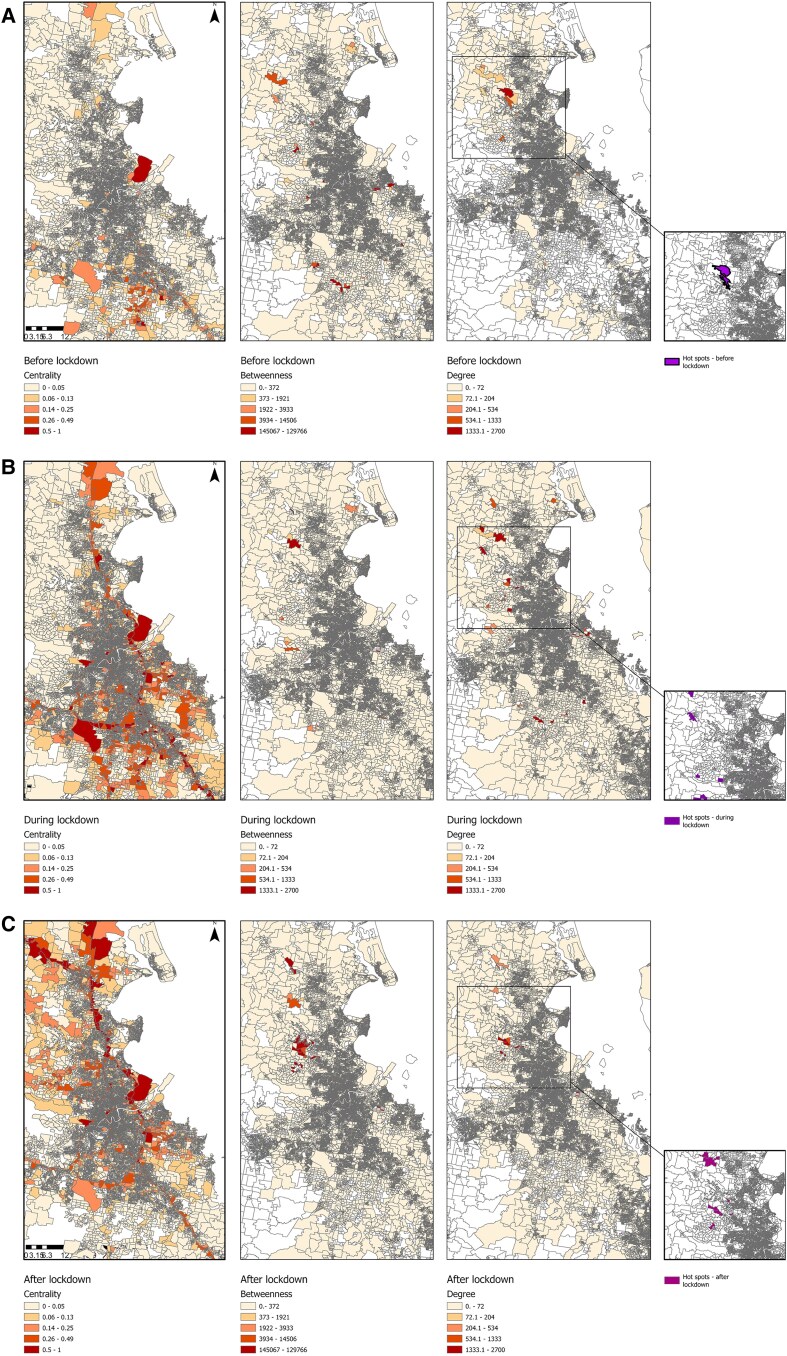
Centrality, betweenness, and degree measures for each period, before (*A*), during (*B*), and after (*C*) lockdown. In purple are represented the mesh blocks that were identified as clusters for each period.

We mapped the value of the network measures for each mesh block and each period (Figure 6). For the prelockdown period, the mesh blocks with the higher centrality measures were mostly located in the north of our study area. In terms of centrality measures across the whole period, mesh blocks with main motorways and the mesh block of the airport had higher centrality measures. Only a small number of mesh blocks have high values for betweenness and degree, indicating limited importance in terms of movement connectivity and flow and limited connectedness between mesh blocks.

### Behavioral Changes and RRV Risk During Pandemic Lockdown

Lockdowns resulted in more time spent in green areas, potentially increasing exposure to mosquitos. The average amount of time people spent in green areas during the prelockdown periods was 81.9 minutes (SD, 219 minutes), or 11.4 minutes per week; during lockdown the mean (SD) was 146 (797) minutes, or 20.5 minutes per week, and after lockdown it was 185 (682) minutes, or 22 minutes per week. We divided the total time (in minutes) spent in each green area and divided that by the number of people who visited the area. We found that the mean time (SD) spent in green areas was 52.2 (184) minutes before lockdown, 55.5 (127) minutes during lockdown, and 81.5 (291) minutes after lockdown. We determined the most visited parks for each period (Supplementary materials). Summarizing the 3 periods together, the most popular park visited was 7th Brigade Park, followed by White Hill Reserve (Supplementary Figure 3).

### Cross-correlation Insights

Our cross-correlation results did not show a significant association between number of weekly RRV notifications and the distance walked or time spent outdoors per week (Figure 4). While a higher number of notifications occurred in week 17, the average walk distance remained similar between weeks 6 and 19. Similar patterns happened between the number of notifications per week and the average walk time spent in green areas, where the time spent outdoors slightly increased between weeks 16 and 19. The lockdown started in week 12 and finished in week 17.

## DISCUSSION

Our study leverages an unusual scenario: the 2020 RRV outbreak coinciding with the initial COVID-19 lockdown in Brisbane. Our findings reveal several interesting associations. First, the RRV peak notifications occurred relatively late in 2020. Second, our model estimated time-varying nonlinear effects of key environmental and network predictors on rates of RRV, with network betweenness becoming a particularly important predictor during the lockdown period. Finally, there was a noticeable rise in the time people spent outdoors in green areas during and after lockdown.

These lockdown observations offer a unique backdrop for our future vector surveillance, a critical component of controlling mosquito-borne disease. However, routine surveillance often fails to monitor critical indicators adequately [20]. Predicting infected vector locations is challenging, especially in urban areas at the start of the season. The disease's incubation period allows exposure across various settings before infection reports. Due to the COVID-19 lockdown in Brisbane, while stay-at-home orders restricted community movement, we expected less interaction with rural and wildlife areas, suggesting that people were exposed to the virus within the city. This supports the idea that RRV can be maintained and transmitted within the human population, potentially downplaying the role of wildlife in RRV transmission.

The 2020 RRV outbreak data, shows a peak in notifications between April and May, which contrasts with previous studies that typically report rising RRV notifications in February, peaking between March and April [21]. Seasonal drivers of RRV transmission include temperature, relative humidity, and rainfall with variations between inland and coastal regions influenced by tidal variability [10, 11, 22]. For example, Hu at al [2] demonstrated that 85% of the variance in the RRV transmission correlated with rainfall. Rainfall, along with mosquito density, emerged as one of the strongest predictors for RRV transmission, with a lag of 1–2 months between rainfall and RRV incidence.

Our findings revealed a 5-week lag between peak rainfall and the subsequent rise in RRV notifications, suggesting that apart from weekly rainfall totals or average maximum temperature there may be additional factors influencing exposure to RRV-infected mosquitos in the areas with reported higher incidence of RRV notifications. Notably, low densities of mosquitoes were reported before the 2020 outbreak [23]. However, unravelling the complexity of RRV outbreaks requires considering region-specific and seasonal variations in key variables [24]. For instance, Brisbane in February 2020 (a month before COVID-19 lockdown was mandated), was affected by intense rainfall and flooding [16]. These unique events may have disrupted the usual link between rainfall and RRV.

Interestingly, despite the role of water bodies in mosquito breeding, no link was found between RRV notifications and proximity to water during the 2020 outbreak. This result diverges from prior studies linking closer proximity to mosquito habitat with increased RRV notifications [25]. However, entomological evidence indicates that while *Aedes* mosquitoes stay within 500 m of their larval habitat, *Culex* mosquitoes can disperse up to 3 km [26, 27]. Although a positive relationship between mosquito density and RRV notification has been observed in Brisbane [28], our study suggests that additional factors were at play.

Community behavior, especially mobility patterns, may have played an important role in the dynamics of RRV transmission during the 2020 outbreak over and above that of weather variability. Indeed, our findings reveal a significant cross-correlation between average time spent outdoors and the weekly number of notifications, as well as between average walk distance and the RRV notifications. Interestingly, the peak of average outdoor time (as depicted in Figure 4) occurred 1 week before the RRV notification peaks (at the transition between the during lockdown and after lockdown). Considering the incubation period of RRV (7–9 days) [3], this temporal alignment suggests that outdoor exposure may have been a more relevant risk factor for RRV transmission during this specific outbreak than the distance to water bodies or parks.

In terms of environmental variables, we found that areas with a higher vegetation cover were associated with higher rates of RRV cases in the prelockdown period. The positive linear association for the that period could be due to fewer RRV cases during this period, as the peak of notification coincided with the lockdown period. However, the relationship was estimated to be weaker and nonlinear during and after lockdown, unlike before lockdown. Previous studies indicated that a higher percentage of vegetation cover can serve as refuge for mosquito and nonhuman vertebrate hosts, both of which play a role in maintaining the RRV in the sylvatic cycle [8].

These findings align with the notion that residing closer to potential mosquito habitat increases the risk of infection [25]. Indeed, a prior study linked certain vegetation, like wetlands and bushland, to RRV risk in Brisbane [29]. Brisbane's extensive vegetation, including tree-lined streets and backyard greenery, is not all officially designated as green spaces. This includes diverse areas from well-kept parks to sparsely vegetated zones. These green areas might support mosquito breeding sites or sustain RRV hosts. Our analysis revealed no correlation between the number of RRV cases and proximity to green areas, when controlling for vegetation density and the remaining predictors. Interestingly, we observed that areas with dense vegetation reported fewer RRV cases during lockdown.

The lockdown's impact on RRV cases and their link to vegetation cover is likely due to behavioral changes. The nonlinear association between RRV rates and vegetation density was particularly clear during lockdown, with a significant portion of Brisbane's population at home. Moreover, our results show the importance of betweenness during lockdown, surpassing environmental conditions in that highly connected areas were associated with a higher incidence of RRV cases, indicating that population movement changes during lockdown were a determinant risk factor for the 2020 RRV outbreak.

Our results indicate a nuanced relationship between vegetation cover and RRV incidence within clusters whereby vegetation cover was positively associated with RRV incidence only up to about 50% coverage, after which this association diminished. This finding underscores the complex interplay between environmental factors and disease transmission. After the lockdown period, residents in RRV clusters likely ventured further into green spaces, where infected mosquitoes are found, once travel restrictions eased. This phenomenon can be attributed to sociological behaviors occurring because of state-mandated public health orders during the COVID-19 pandemic, which deserve further investigation.

The role of human movement is a key factor in understanding the dynamic of vector-borne disease, however, it has been understudied [30]. While our findings indicate that the population from RRV clusters identified during lockdown had an increased average time spent in green areas, these results come with significant variability. This finding aligns with a Brisbane survey finding that approximately 36% of respondents increased their use of urban green spaces [31]. Despite various species testing virus positive, their transmission role is still unclear [9]. It is possible that urban wildlife played a relevant role in the RRV outbreak. In Brisbane, rich in opossums and urban-rural macropods, both species have tested virus positive [28].

Given the limited travel during lockdown, local mosquitos were likely outbreak vectors. The main vector, *Culex annulirostris,* could have been widespread despite low numbers [23]. Indeed, recent evidence indicates human-mosquito contact alone might sustain virus transmission. This hypothesis is supported by the discovery that humans had a moderate-to-high physiological competence with respect to RRV [9]. Urban wildlife likely initiated the 2020 RRV outbreak, which was then accelerated by subsequent mosquito-to-human transmission from infected individuals. Our findings highlight the need to include human mobility and outdoor time in future disease outbreak prediction.

Limitations must be carefully considered when interpreting the study results. During the prelockdown period for COVID-19, movement records were limited due to our focus on cluster areas. The clusters during this period were aggregated in the north of the study area, and therefore the results can be biased. Mobile phone tracking data, used as a proxy for actual RRV exposure, only provides general population movement insights. Furthermore, our study's scope was restricted to a single outbreak during COVID-19 lockdowns, potentially limiting its applicability to past and future RRV outbreaks without movement restrictions.

Our study offers insights for enhancing mosquito surveillance for RRV exposure by focusing on areas where people spend significant outdoor time. We found a correlation between these areas, especially those with high connectivity mesh blocks, and increased RRV notifications. Adapting protocols and mosquito control in these areas can improve RRV prevention and public health protection.

## Supplementary Data

Supplementary materials are available at *The Journal of Infectious Diseases* online (http://jid.oxfordjournals.org/). Supplementary materials consist of data provided by the author that are published to benefit the reader. The posted materials are not copyedited. The contents of all supplementary data are the sole responsibility of the authors. Questions or messages regarding errors should be addressed to the author.

## Supplementary Material

jiae578_Supplementary_Data

## Notes

***Acknowledgments.*** We acknowledge members of the Mosquito and Arbovirus Research Committee (MARC) for comments, feedback, and funding of this research.

***Author contributions*.** Conceptualization: J. M. D., D. S. M., D. F., and R. J. S. M. Formal analysis: T. P., D. B., N. J. C., and S. A. Methodology: T. P. and R. J. S. M. Supervision: R. J. S. M. Writing original draft: T. P. Review and editing: N. J. C., G. D., J. M. D., D. S. M., D. F., and R. J. S. M.

***Financial support*.** This work was supported by the HEAL (Healthy Environments and Lives) National Research Network, which receives funding from the National Health and Medical Research Council Special Initiative in Human Health and Environmental Change (grant 2008937), and by the Mosquito and Arbovirus Research Committee (MARC) .
