# Supplementary material for: Ascertainment of Community Exposure Sites to Ross River Virus During the 2020 Outbreak in Brisbane, Australia
Source: J Infect Dis. 2024 Nov 26;231(3):e501–10. doi: 10.1093/infdis/jiae578 (PMC11911798; doi:10.1093/infdis/jiae578)
Supplement: jiae578_Supplementary_Data [file jiae578_supplementary_data.docx]

Supplementary material

Ascertainment of community exposure sites to Ross River virus during the 2020 outbreak in Brisbane, Australia

Tatiana Proboste, Damber Bista, Nicholas J Clark, Sahil Arora, Gregor Devine, Jonathan M Darbro, Deena S Malloy, Daniel Francis, Ricardo J. Soares Magalhães

Methods

RRV data source

This project (ID 71859 LNR/2021/QRBW/71859) received ethical approval from Royal Brisbane and Women's Hospital (HREC 71859), research ethics ratification from The University of Queensland (2021/HE000937), and Public Health Act authorisation to request health information held (PHA 71859).

The data obtained from Notifiable Condition System included the following variables: notification ID, notifiable disease description (limited to RRV only), notification date, onset date, which is an estimate, based on the reported onset of illness at the time of presentation to a medical clinic, month, age at onset, sex, street number and name, postcode, locality, public health unit (limited to Metro North and Metro South).

Environmental data

We retrieved environmental data known to affect mosquito density from the Queensland Government longpaddock website (https://www.longpaddock.qld.gov.au/) using the R package cropgrowday (Baker P, 2021). These data included a raster file of rainfall per week (mm) and average maximum temperature (°C) per week. We also included in our analyses the distance to water bodies (Wetland data - Queensland, 2019). To calculate the distance of the water body, we first maintained only the water bodies that were classified as riverine, palustrine, and lacustrine, as these are the most potential sites where mosquitoes might breed, and then we calculated the Euclidean distance to create a raster. To create the green areas layer, we combined information from Brisbane City Council (Brisbane City Council) and QLD protected areas (Qldspatial) and calculated the Euclidean distance to create a raster. The combined information gave us an estimate of the green areas for the RRV-affected region. Both distance calculations were done in ArcGIS Pro 2.9.5. For the vegetation density, we used the foliage projective cover, which represents the percentage of ground area occupied by

the vertical projection of the foliage of woody vegetation (Queensland government). For the analyses, we calculated the mean of all environmental data to the mesh block level.

Population movement data and data processing steps

All data was further deidentified by aggregating GPS signals to the mesh-block level to be in geographic concordance with the RRV NOCS data. We performed a thorough data cleaning process on the GPS dataset, removing duplicates and retaining only records above 5 minutes duration. We included only IDs that have more than the median for the highest point to be assumed as a household (we kept IDs ≥4, given the median of 4). We assumed that the location with the highest number of records would correspond to their household's mesh-block.

Association between RRV notification incidence, environmental data and population movement network measures

Network measures: We utilized Kleinberg’s authority centrality as our centrality measure, which identifies the most important nodes in each network. A node is classified as an authority if it is connected to by hubs, whereas it is identified as a hub if it connects to authorities. Betweenness represents the shorter path in the network and degree assigns an importance score based on the number of unique links by each node (in our case the mesh-block).

The GAM was built using the number of RRV cases in each mesh block in each time period as our outcome variable. Cubic regression smooths of green areas, distance to water and vegetation density were included with k = 10 knots for each effect. Tensor product smooths of latitude and longitude were included to capture any un-modelled spatial autocorrelation (using cubic regression marginal smooths of latitude and longitude with k = 20 knots each). For each smooth function, we allowed the degree of smoothness and the overall shapes of the nonlinear functions to vary over time using the ‘by’ argument in the s() and te() functions in the mgcv R package [18]. All smooths were fully penalized (by using the select = TRUE argument in the bam() function in mgcv) to ensure that any variables with limited support for inclusion in the model were automatically regularized to flat functions. We included the log(population) of each mesh-block as an offset variable.

Results

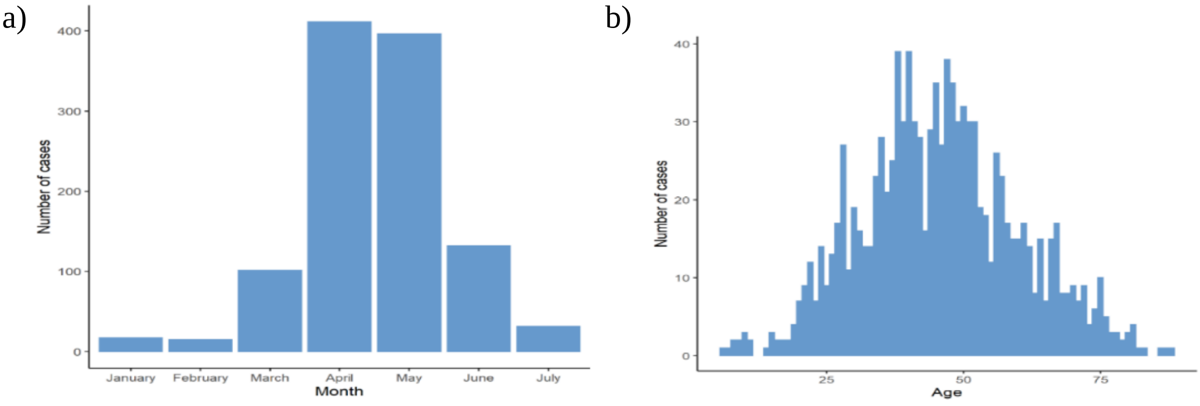

Figure S1. a) Monthly distribution of incidence of RRV notifications across Metro North and Metro South Public Health Units between January and July 2020; b) Age-incidence profile of RRV notifications across Metro North and Metro South Public Health Unit between January and July 2020.

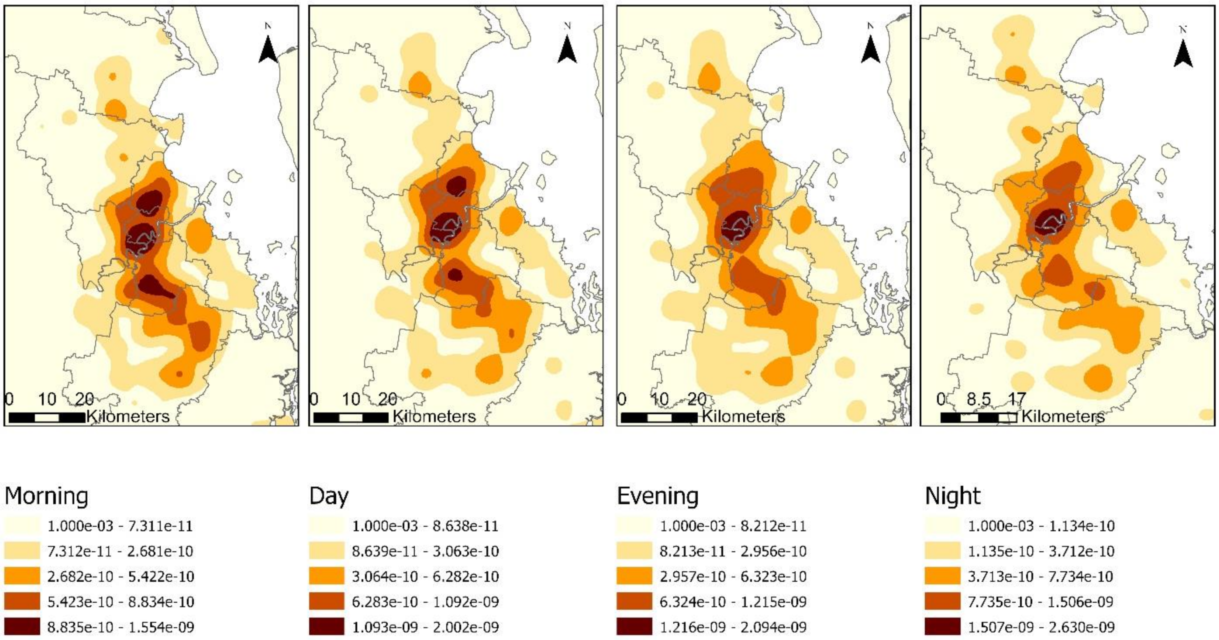

Figure S2. Kernel density of the people's movement across different times of the day.

Relationship between population movement network and landscape visited

For the period of pre-lockdown, most of the time spent walking outdoors overlapped with the Guyatt park (St Lucia), during-lockdown 7th Brigade Park (Chermside), and after-lockdown, Whites Hill Reserve (Camp Hill). If we summarise the 3 periods together, the most popular parks visited was 7th Brigade Park followed by White Hill Reserve (Fig. S3).

a)

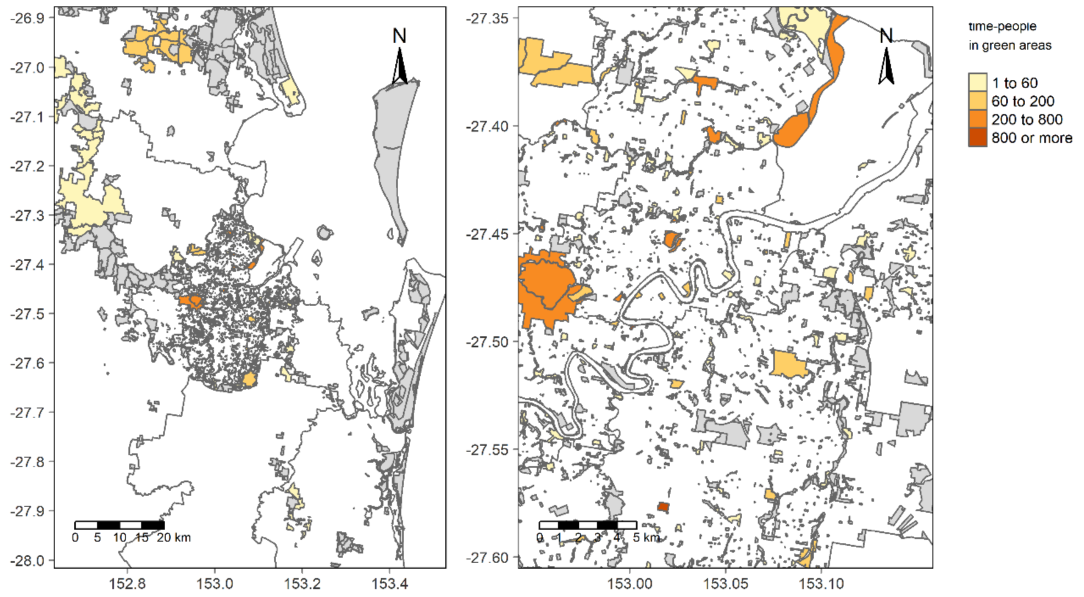

b)

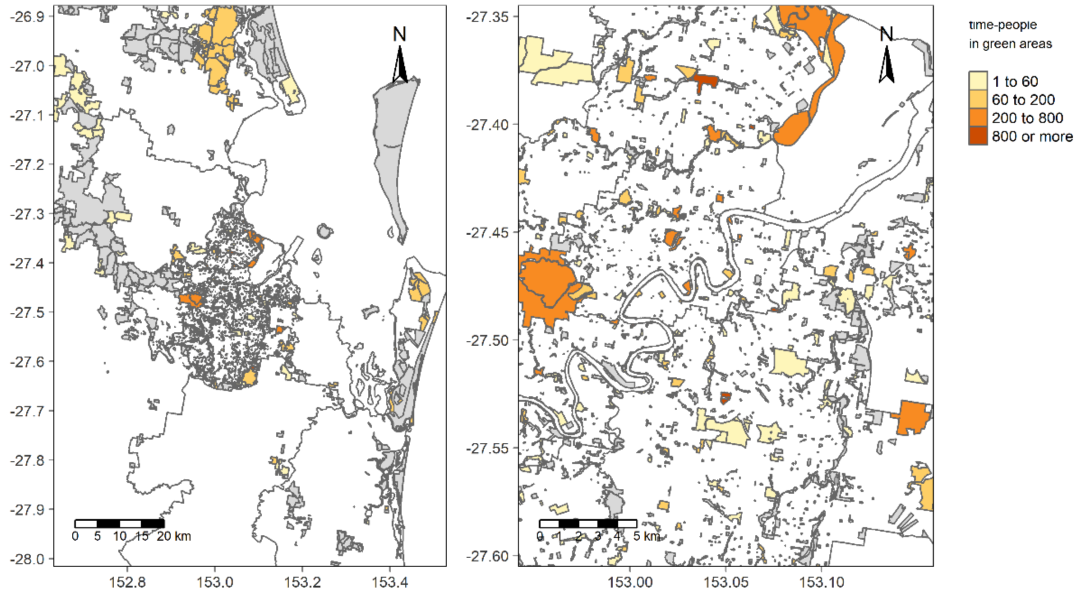

c)

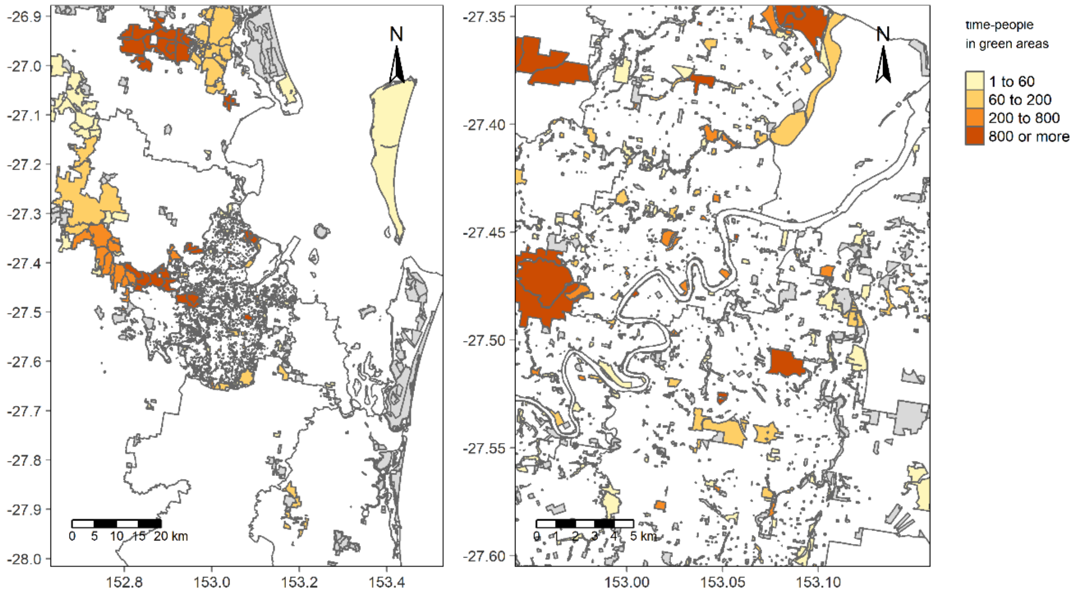

Figure S3. Areas where people spend time walking in green areas during each period (in minutes). a) pre lockdown, b) during lockdown, c) after lockdown.

0

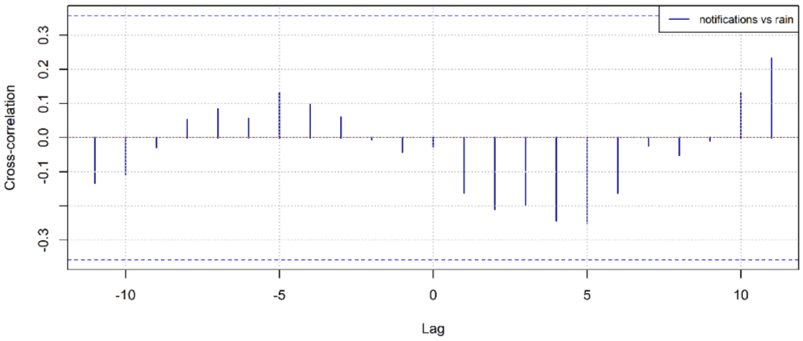

b)

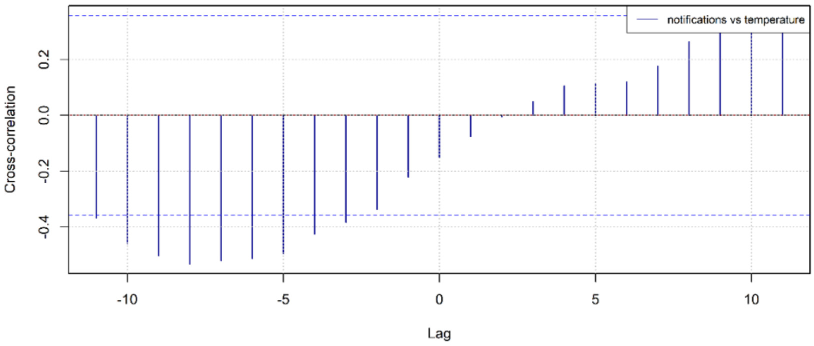

c)

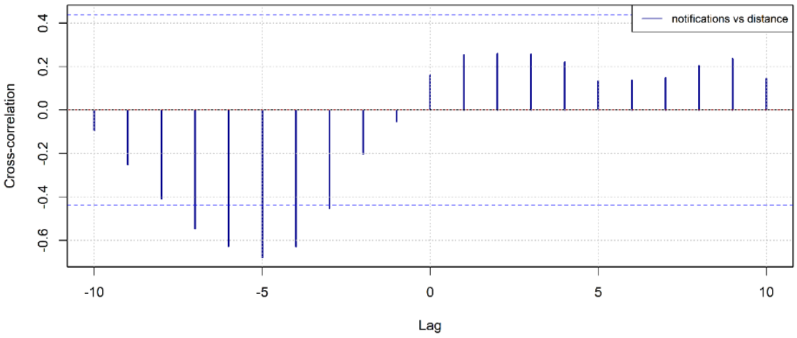

d)

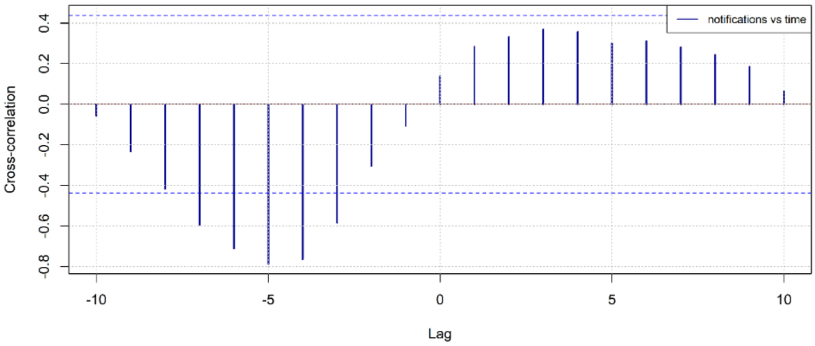

Figure S4. Cross-correlation plots for the a) RRV notifications and total rain (mm), b) RRV notification and average maximum temperature, c) RRV notification and average walk distance and d) RRV notification and average time outdoor

79

Pre-lockdonw During-lockdown After-lockdown

a)

b)

c)

4

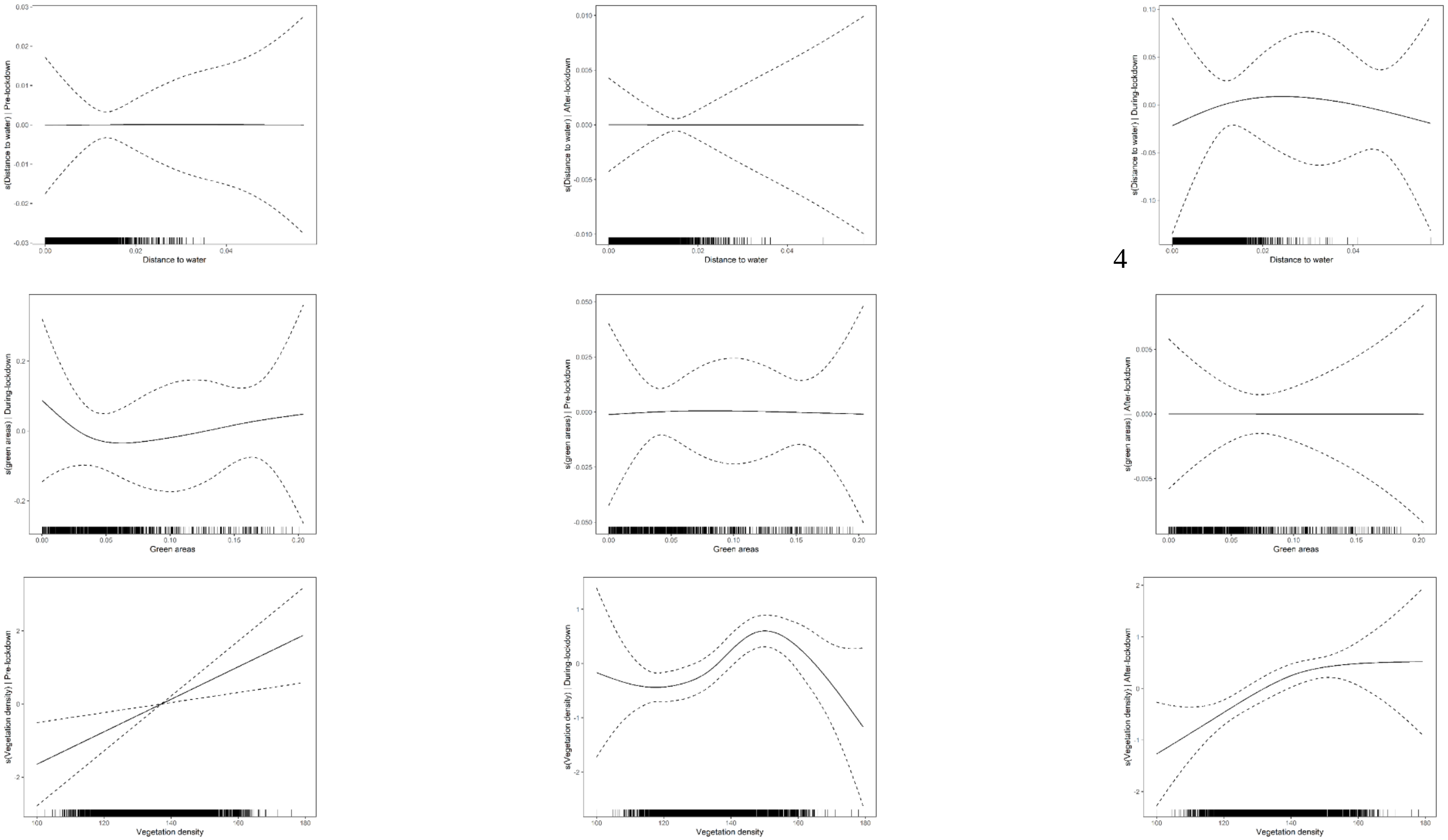

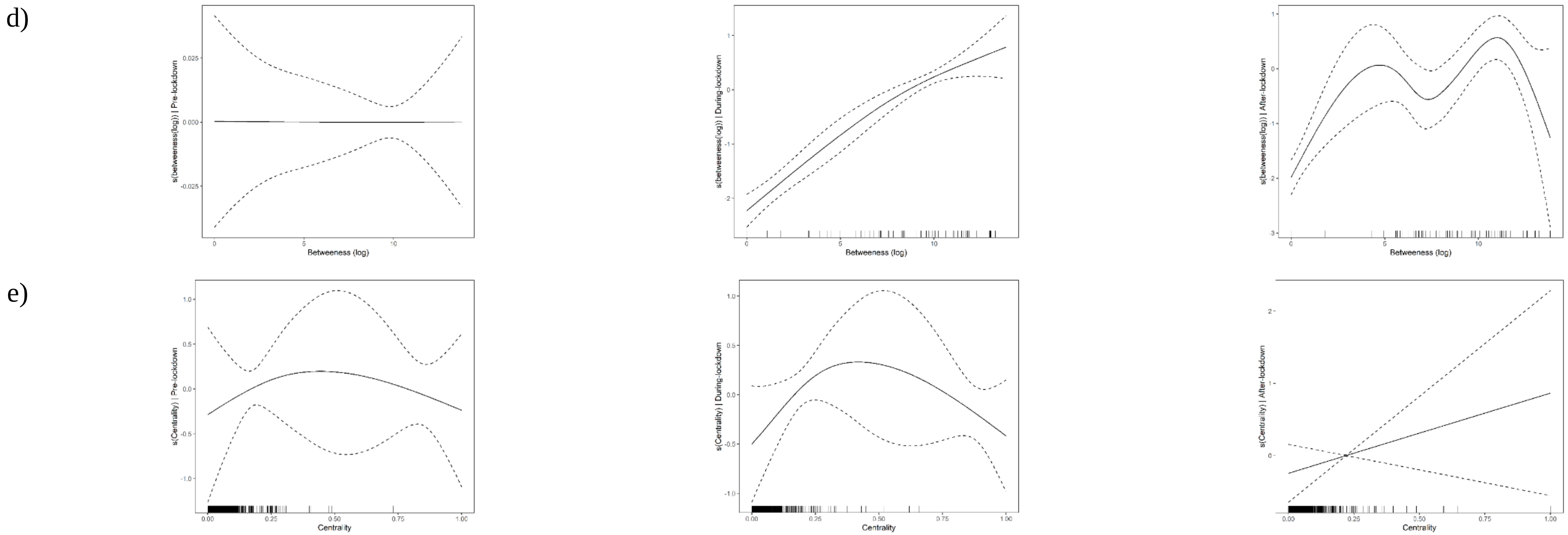

Figure S5. Plots of smooth effects of log(number of RRV) cases predicted from a generalized additive model when responding variables were a) distance to water, b)distance to green areas, c) vegetation density, d) Betweenness, e) Centrality.

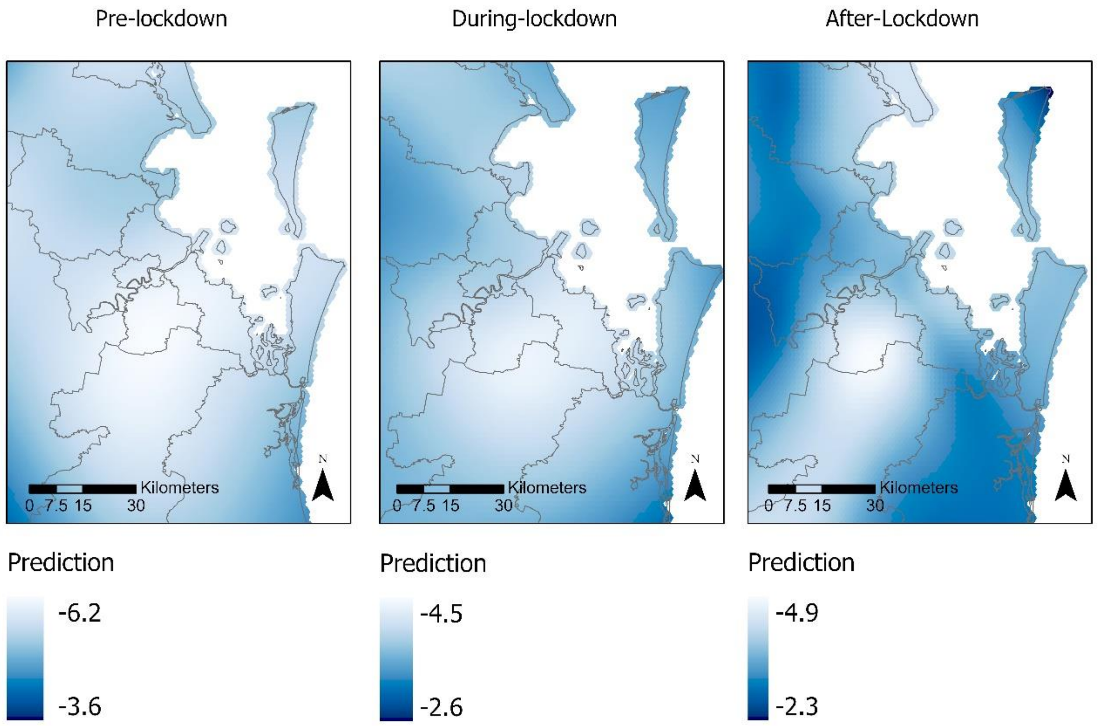

Figure S6. Spatial terms over time. Distribution of the prediction values based on the log(number of cases) for the pre-lockdown, during-lockdown and after-lockdown

References

Baker P, M. M. (2021). *cropgrowdays: Crop Growing Degree Days and Agrometeorological Calculations*.

Brisbane City Council. https://www.data.brisbane.qld.gov.au/

Qldspatial. *Queensland Spatial Catalogue - QSpatial*. https://qldspatial.information.qld.gov.au/catalogue/custom/index.page

Queensland goverment. *Foliage Projective Cover (FPC)*. https://www.data.qld.gov.au/dataset/landsat-foliage-projective-cover-queensland-2014

Wetland data - Queensland Version version 5). (2019). https://qldspatial.information.qld.gov.au/catalogue/custom/search.page?q=%22Wetland%20data%20-%20version%205%20-%20wetland%20areas%20-%20Queensland%22
